# Deletion of PEA-15 in mice is associated with specific impairments of spatial learning abilities

**DOI:** 10.1186/1471-2202-10-134

**Published:** 2009-11-16

**Authors:** Joe W Ramos, David A Townsend, Dawn Piarulli, Stefan Kolata, Kenneth Light, Gregory Hale, Louis D Matzel

**Affiliations:** 1 Cancer Research Center of Hawaii, Department of Natural Products and Cancer Biology, University of Hawaii at Manoa, 651 Ilalo Street, Honolulu, HI 96813, USA; 2 Department of Psychology, Program in Behavioral Neuroscience, Rutgers, The State University of New Jersey, Busch Campus, Piscataway, NJ 08854, USA; 3 Department of Cell Biology and Neuroscience, Rutgers, The State University of New Jersey, 604 Allison Road, Piscataway, NJ 08854, USA

## Abstract

**Background:**

PEA-15 is a phosphoprotein that binds and regulates ERK MAP kinase and RSK2 and is highly expressed throughout the brain. PEA-15 alters c-Fos and CREB-mediated transcription as a result of these interactions. To determine if PEA-15 contributes to the function of the nervous system we tested mice lacking PEA-15 in a series of experiments designed to measure learning, sensory/motor function, and stress reactivity.

**Results:**

We report that PEA-15 null mice exhibited impaired learning in three distinct spatial tasks, while they exhibited normal fear conditioning, passive avoidance, egocentric navigation, and odor discrimination. PEA-15 null mice also had deficient forepaw strength and in limited instances, heightened stress reactivity and/or anxiety. However, these non-cognitive variables did not appear to account for the observed spatial learning impairments. The null mice maintained normal weight, pain sensitivity, and coordination when compared to wild type controls.

**Conclusion:**

We found that PEA-15 null mice have spatial learning disabilities that are similar to those of mice where ERK or RSK2 function is impaired. We suggest PEA-15 may be an essential regulator of ERK-dependent spatial learning.

## Background

PEA-15 is a 15 KDa phosphoprotein that regulates both ERK MAP kinase and death receptor apoptosis pathways [1-3]. PEA-15 regulates ERK MAP kinase by binding directly to it and maintaining active ERK in the cytoplasm [2]. By this mechanism PEA-15 alters the transcriptional response to growth factors that stimulate the ERK MAP kinase pathway. PEA-15 can also bind and alter activation of the kinase RSK2, which is a substrate of ERK [4]. Indeed PEA-15 acts as a scaffold to enhance ERK activation of RSK2 and subsequent CREB transcription [5]. Moreover, in PEA-15 null cells (including astrocytes) RSK2 activation is impaired [5]. PEA-15 is expressed in both neurons and astrocytes throughout the brain [6]. A physiological role of PEA-15 in the brain has not been determined though it has been implicated in diverse pathological conditions including type II diabetes [7,8], glioma [9,10], and breast cancer [11,12].

The ERK MAP kinase pathway is involved in learning and memory. In particular, it is implicated in spatial learning and fear conditioning [13]. Both of these processes, in some forms, are thought to be dependent on the hippocampus. At the molecular level, blocking ERK activity impairs spatial learning [14,15] and long term potentiation (LTP) [16]. Activated ERK translocates to the nucleus where it initiates transcription by phosphorylating transcription factors including ELK-1 and c-Fos. In the cytoplasm ERK may phosphorylate several substrates including cytoskeletal proteins like stathmin as well as other kinases such as RSK2 [17]. RSK2 is a complex kinase containing two kinase domains. Like ERK, activated RSK2 can translocate into the nucleus where it activates transcription factors including c-Fos and CREB [18]. Moreover, RSK2 is expressed at high levels in structures of the brain such as the hippocampus, neocortex and purkinje cells that are associated with cognitive function and learning [19]. Mutations in RSK2 cause Coffin-Lowry syndrome (CLS) [20]. CLS patients suffer from cognitive impairment and skeletal deformations [21]. Furthermore the cognitive impairment associated with CLS correlates with a reduction in RSK2 activity [22]. Similarly, mice in which RSK2 has been deleted have impaired spatial learning and reduced exploratory behavior [23]. One target of RSK2 activity is the transcription factor CREB. There is a growing literature describing the importance of CREB in Long Term Potentiation (LTP) and learning [24].

Because PEA-15 can regulate both ERK and RSK2 we assessed PEA-15 knockout (KO) mice in a series of tests that measure learning, sensory/motor function, and stress reactivity. We found that deletion of PEA-15 protein in C57BL/6J mice causes specific defects in spatial learning abilities.

## Results

**Figure 1:**
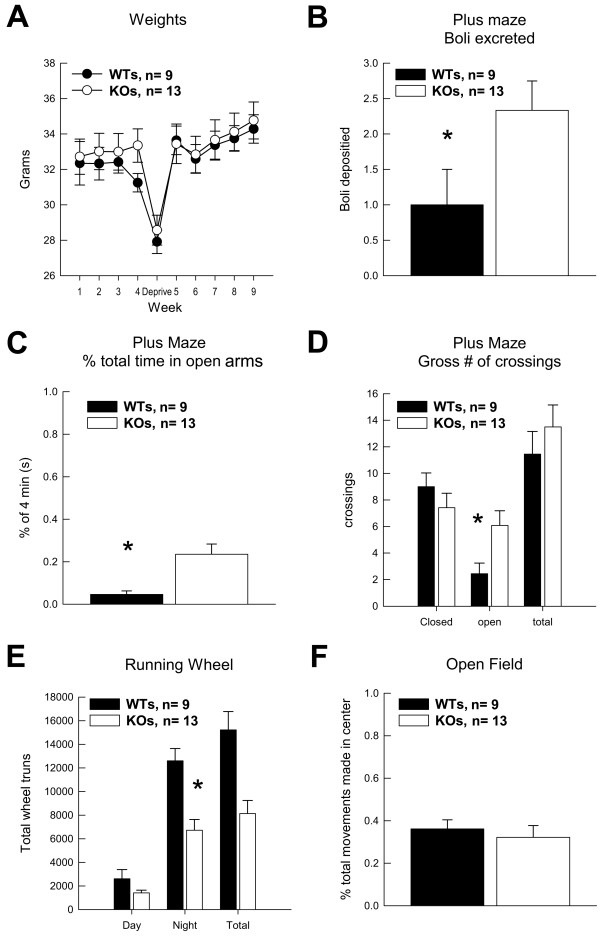
**PEA-15 knockout mice exhibit irregular reductions in activity and higher stress reactivity**. (A) Average weight per group across the nine weeks of experimentation is depicted. (B) Stress induced boli deposited in the Elevated Plus Maze is shown. (C) Average time per group spent in the open arms of the Elevated Plus Maze during the 4 min session is shown. (D) Average number of crossings into open and closed arms in the Elevated Plus Maze is depicted. (E) Shown is the average percent of time per group spent in the center (stressful) quadrants of the Open Field. (F) Total average revolutions made in the Running Wheel task for each group is shown. Data are shown as mean values ± SEM; n values are indicated. "*" indicates a *P *value < 0.05.

PEA-15 is expressed at high levels throughout the nervous system. PEA-15 null (KO) mice have been reported to have normal brain sizes and astrocyte numbers and to be born in roughly normal ratios [3]. No differences in brain morphology have been identified in these mice. PEA-15 is known to modulate both ERK and RSK2 signal transduction and may therefore affect cellular function in the nervous system. We therefore designed a series of tests to determine if PEA-15 KO mice suffered from sensory-motor, behavioral, or learning deficiencies compared to wildtype (WT) controls. KO mice did not differ from the WT mice in their body weight at any time during the experiments (Figure 1A). Both groups reacted similarly to food deprivation, shedding approximately 4 to 8% of gross body weight during deprivation. Additionally, both KO and WT mice recovered pre-deprivation weights at the same rate and magnitude.

### Stress and activity tasks

In elevated plus maze tests KOs (n = 13) deposited more bolli in the maze than WTs (n = 9), [F_(1,20) _= 4.27; *p *< 0.05] (Figure 1B) suggesting that exploration by KOs into the open arms of the plus maze increased these subjects' stress reactivity. However, the stressful nature of the open arms did not diminish KOs exploration relative to WTs. KOs spent more time than WTs in the open arms of the elevated plus maze [F_(1,20) _= 10.79; *p *< 0.05] (Figure 1C). This difference was not due to an increase in basal activity, as both groups made a comparable amount of arm crossings (Figure 1D). However, KOs chose to enter open arms significantly more often than their WT counterparts did [F_(1,20) _= 6.25; *p *< 0.05]. There was also a trend for KOs to make a first open arm entry more quickly than WTs. Therefore KOs tended to exhibit higher levels of anxiety or fear with a concomitant increase in exploration of the maze.

An alternative test of anxiety and exploration is the Open Field test. In this test KO and WT mice exhibited similar numbers of total movements in the open field (Figure 1F). However in contrast to the elevated plus maze findings, KO mice did not differ from WTs in the amount of time spent in 'open' stressful quadrants relative to 'safe' wall quadrants. Exploration of the open quadrants of an open field can be used as an index of an individual mouse's inclination for novelty seeking. In this experiment, PEA-15 KO and WT mice did not differ in their proclivity to engage new environments.

In the Black/White Preference test, mice have a propensity to spend more time in the black, dimly lit half of the preference chamber presumably because they find this chamber less stressful than the well-lit white half and because animals are introduced into the apparatus on the black side. In this test, both KO and WT groups spent more of the total time during testing in the black/dim side of the chamber as is normal. However, KO mice made their initial crossing into the white side of the chamber more slowly than WT mice [F_(1,20) _= 5.34; *p *< 0.05] and spent significantly less time in the white well lit side [F_(1,20) _= 5.76; *p *< 0.05] (data not shown). This difference was not due to differences in activity between the groups, since both made a similar number of crossings between the two sides of the apparatus. This test is further evidence that KO mice suffer increased levels of anxiety or fear.

As a separate measure of fear we examined KO mouse performance in the Straight alley/Escape test. The startle stimulus (bright light combined with increased air flow) used during this test typically elicits rapid running (an "escape" response), which serves as an index of fear. Though the escape distance for PEA-15 null mice was only 63% of that for wildtype mice (*X *= 225.8 cm, 361.2 cm respectively), this difference was not significant [F_(1,20) _= 2.86; *p *< 0.10]. Groups did not differ in their latency to explore the escape straight alley, nor did they show a preference for either end of the apparatus (start box or open end). Hence, KO mice do not appear to suffer increased fear as measured in this test. In total, the differences noted above for the elevated plus maze and the black/white preference test suggest a pattern of behavior in KO mice (i.e., an *increase *in stress-provoking behaviors in the elevated plus maze and a *decrease *in these behaviors in the black/white box) that does not reflect a consistent pattern of differences in stress or anxiety related behaviors. Thus these differences may reflect other, unidentified causes.

To assess potential differences in physical activity that may contribute to differences in these tests, the time spent by KO and WT mice on a running wheel was assessed. PEA-15 null mice utilized the running wheel significantly less than wildtype mice during the 48 hours of the test [F_(1,20) _= 18.27; *p *< 0.001]. This difference was not due to less running behavior during the light cycle, when all mice spent significantly less time running, but was due to KOs spending significantly less time running during the night cycle [F_(1,20) _= 14.59; *p *< 0.001] (Figure 1E). Thus KO mice were generally less active than their WT counterparts.

### Sensory Motor tasks

**Figure 2:**
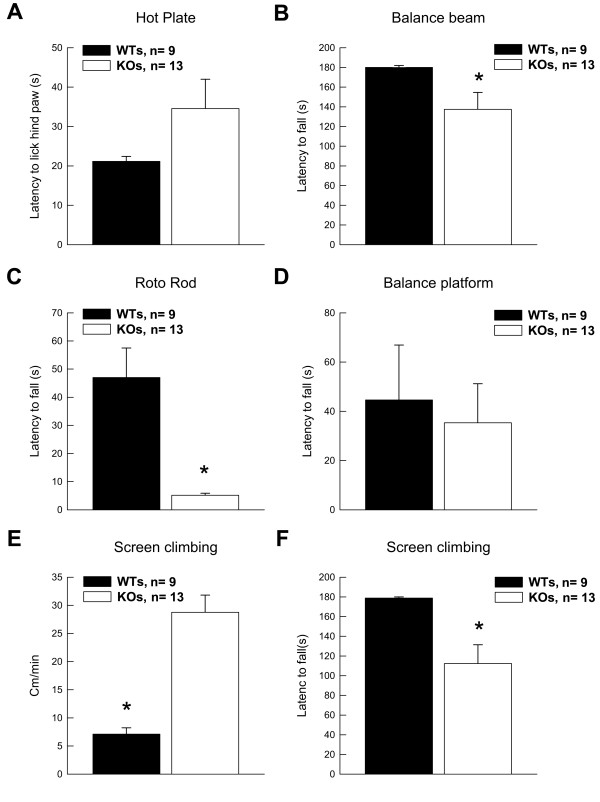
**Sensory and motor tests reveal that PEA-15 Knockout mice have reduced forepaw strength**. (A) Groups average latencies to lick hind paws in the pain sensitivity measure are shown. (B) Groups averages to fall off the apparatus in the Balance Beam task are shown. (C) Groups averages to fall off the apparatus in the Roto Rod test are shown. (D) Groups averages to fall off the apparatus in the Balance Platform task are shown. (E) Average groups movements made while on the Screen Climbing apparatus are shown. (F) Groups averages to fall off the Screen Climbing apparatus are shown. Data are shown as mean values ± SEM; n values are indicated. "*" indicates a *P *value < 0.05.

To determine whether PEA-15 null mice have normal responses to pain we assessed their sensitivity to heat by measuring their latency to lick or shake a hind paw when placed on a hot plate. There was a tendency for PEA-15 KO (n = 13) mice to react slower to the painful stimulus than WT mice (n = 9) [F_(1,20) _= 3.49; *p *< 0.07] (Figure 2A). Differences in pain reactivity could possibly affect learning tasks that entail painful reinforcement, such as associative fear conditioning.

We next examined if KO mice' strength or motor function are impaired. PEA-15 KO mice performed worse than WT mice in tests that assessed grip strength or coordination, measured by latency to fall from an apparatus in three sensory/motor tasks (Figure 2B-F). Specifically, KOs (n = 13) were impaired compared to WTs (n = 9) in successful completion of the Balance Beam test [F_(1,20) _= 4.69; *p *< 0.05] (Figure 2B), the Roto-Rod test [F_(1,20) _= 20.87; *p *< 0.001] (Figure 2C), and movements during the Screen Climbing test [F_(1,20) _= 8.26; *p *< 0.01] (Figure 2E, F). In all three tests, KOs fell off more quickly. These deficits appear to be unrelated to balance or coordination, as both groups performed identically on the Balance Platform [F_(1,20) _= 0.12; n.s.] (Figure 2D).

### Learning Tasks

**Figure 3:**
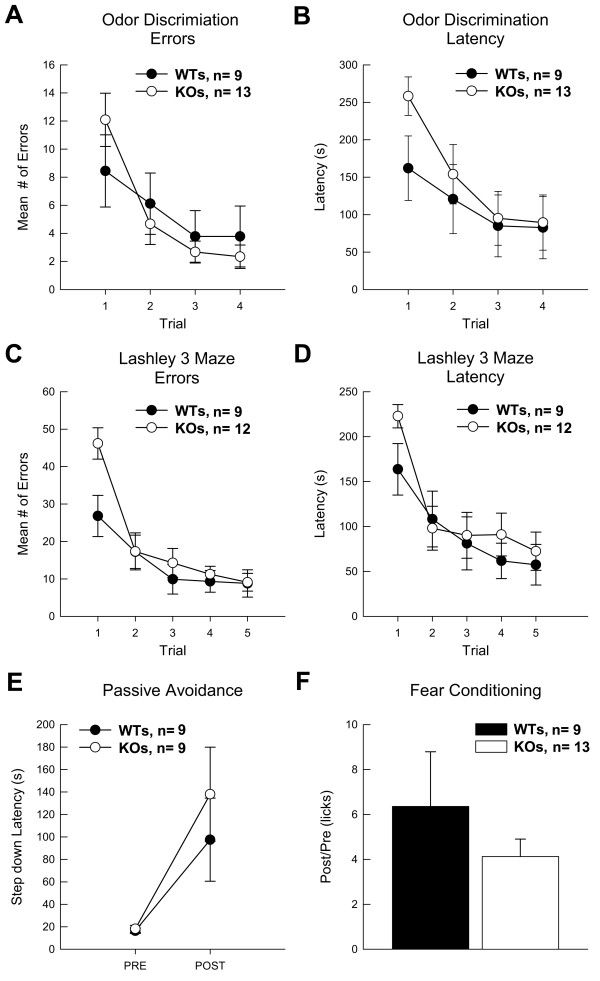
**Wildtype and PEA-15 Knockout mice did not differ significantly in acquisition during non-spatial learning tasks**. (A) Average number of errors committed during acquisition of the Odor Discrimination task are depicted. (B) Latencies to find the reward in the Odor Discrimination task are shown. (C) Average number of errors committed during acquisition in the Lashley 3 Maze is shown. (D) Latencies to transverse the maze and find the reward in the Lashley 3 Maze are depicted. (E) Passive avoidance is measured as step-down latencies before (pre) and after (post) training in the Passive Avoidance task. Four mice, two from each group, were removed from this test because their pre-training latencies did not meet criteria: that is they did not step down in the maximum 300 seconds allowed during training. (F) Conditioned Fear is expressed as a ratio of the latency to make 25 licks before (pre) and after (post) CS (tone) presentation subsequent to associative fear conditioning. One mouse was not included in the fear conditioning data because of an apparatus recording failure. Data are shown as mean values ± SEM; n values are indicated.

In odor discrimination, KO mice did not differ from WTs during learning (Figure 3A). Both types of mice mastered the task across trials (2-4) [F_(2,38) _= 5.12; *p *< 0.01]. However, KOs (n = 13) were impaired in their mean latency to locate the reinforcer [F_(1,20) _= 4.09; *p *< 0.057] on the first training trial (before any learning could occur) (Fig 3B). KOs also tended to make more errors than WT mice (n = 9) on the first trial (Figure 3A), although this difference did not approach significance. The comparable learning rates exhibited by KO and WT mice suggests that PEA-15 KO mice retain normal olfactory abilities and learning about olfactory stimuli. In the Lashley III Maze, KO mice (n = 12) did not differ from WT (n = 9) mice in acquisition of this task, as measured by either latency to transverse the maze or the number of errors committed in route to the goal box (Figure 3C and 3D). Over trials, the latency of both groups to locate the goal box decreased, as did their errors (i.e., wrong turns or retracing). As in odor discrimination, on Trial 1 (before any learning could have occurred), KO mice recorded longer latencies to find the goal box [F_(1,19) _= 4.21; *p *< 0.05] and committed more 'errors' [F_(1,19) _= 8.17; *p *< 0.01] than WTs.

In these experiments mice learn to suppress movement to avoid the aversive stimuli (bright light and loud oscillating noise). To control for individual basal levels of activity and exploration, step-down latencies were calculated by utilization of a ratio of post training latencies as a function of pre-training latencies. Both KO (n = 9) and WT (n = 9) mice significantly increased their latencies to step down after training [F_(1,16) _= 10.95; *p *< 0.01], but there was no difference between KOs and WTs in performance in this test (Figure 3E).

To assess associative fear conditioning KO and WT mice were exposed to a stimulus (white noise) that terminated with a mild foot shock. The latency of water-deprived mice to complete 25 licks of a water tube during the white noise presentation was used as a measure of whether the mouse had learned to associate the white noise with the shock. Increased latency to complete 25 licks during the noise compared to before (Pre) the white noise in each mouse indicates associative fear conditioning. There was no significant difference between KO (n = 13) and WT (n = 9) mice in this task (Figure 3F). KOs did not differ significantly from WTs in basal drinking rates or in consumption of water during testing

In Reinforced Alternation tests, mice learn to alternate arm choices in an elevated T-maze to obtain food reinforcement. The number of training trials required before an animal learns to consistently alternate arm choices is an index of the rate at which they learn the pattern. Previously, with our adaptation and training procedures young adult CD-1 and BALB/C mice often began to perform without error after 8-10 training trials. However in these experiments with C57BL/6J mice neither KO nor WT mice learned the correct pattern competently (data not shown). During acquisition trials, KOs only performed at chance rates, while WT mice chose the correct arm approximately 65% of the time. Because of the poor performance during reinforced alternation training, representative groups (n = 5) of WT and KO mice were given an additional day of training (12 trials). Though both groups improved correct choices to nearly 75%, there was no mean difference between KOs and WTs (data not shown).

**Figure 4:**
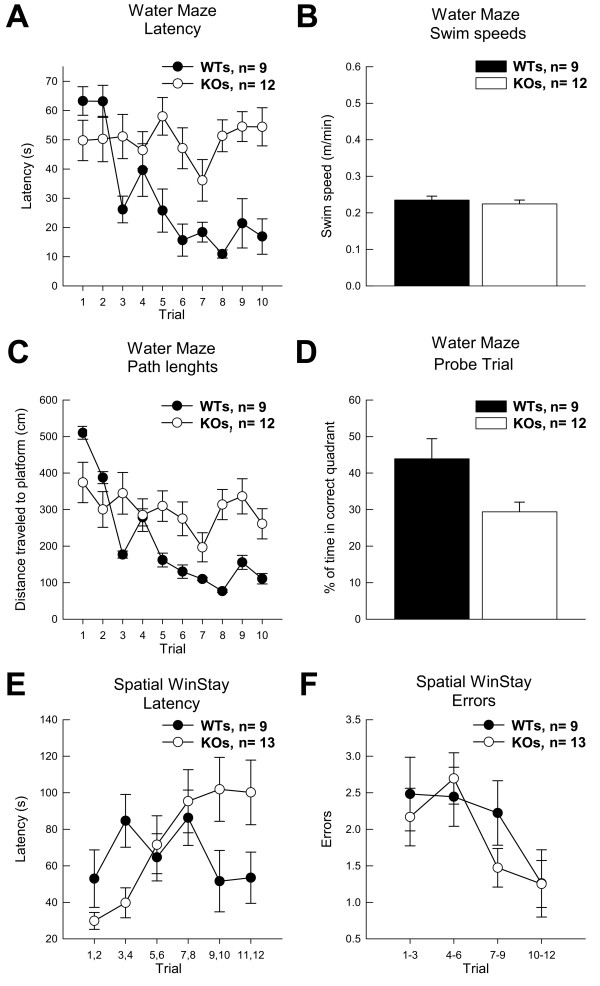
**PEA-15 Knockout mice were impaired compared to Wildtypes in acquisition of tasks with a spatial component**. (A) Average group latencies to locate the hidden platform across trials in the Water Maze are shown. (B) Average swim speeds during early acquisition in the water maze are shown. (C) The group average path length to the hidden platform is shown. (D) Performance during the water maze probe trial is shown. (E) Group average latencies to locate the reward during the Spatial WinStay (plus maze) are depicted. (F) Average errors groups committed during acquisition of the Spatial Win Stay task are depicted. The Trials along the X-axis are collapsed for clarity. Data are shown as mean values ± SEM; n values are indicated.

We next tested PEA-15 null mice in the Morris water maze. Acquisition and performance in the spatial version of the Morris water maze is dependent on an intact hippocampus and is said to indicate the animals' representation of its environment as a "cognitive map" [25,26]. Across the two days of training, KO (n = 12) animals never improved their performance in locating the hidden platform (Figure 4A) and the inability to master this spatial task is in marked contrast to WTs (n = 9) performance [F_(9,171) _= 5.22; *p *< 0.0001]. This difference was not due to reduced strength or activity of the KO mice since all mice had similar swim speeds [F_(1,19) _= 0.20; n.s.] on the first training trial before any learning had occurred (Figure 4B). The path lengths of the KO mice, similar to their latency to find the platform, did not change during training (Figure 4C). Because WTs learned the task, their path-lengths to the platform improved during training [F_(9,171) _= 3.44; *p *< 0.0001]. Not surprisingly, WT controls also out-performed KOs during a probe trial after training [F_(1,19) _= 5.61; *p *< 0.05]. KOs spent equivalent search time in the target quadrant as in the other three quadrants (Figure 4D). This gross impairment was not due to KOs inability to swim, their swim speed, or ability to stay on the platform.

Similar to the water maze, the spatial version of the win/stay task in a plus maze requires animals to utilize a cognitive map or spatial cues. In contrast to the water maze, animals receive a food reward for successfully completing a trial rather than escape from water. In the spatial win/stay experiments KO (n = 13) mice were impaired compared to WTs (n = 9), [F_(1,20) _= 4.81; *p *< 0.05] during the final four trials as measured by latency to navigate the maze and make an arm choice (Figure 4E). While a tendency was observed for KO animals to make more errors in this task (on Trials 7-9, where WT animals underwent clear acquisition), this tendency did not reach statistical significance, [F_(1,20) _= 2.03; ns] (Figure 4F). Since both KO and WT mice reduced errors throughout training, the increased latencies exhibited by KO animals suggest that KO mice might have an increased time latency relative to their WT counterparts

The PEA-15 null mice appeared to be impaired in tasks that can be solved using a spatial strategy (Water maze, Spatial win/stay). To further explore this observation, mice were tested in a modified version of the spatial win/stay task. This test was executed in the same manner as the earlier spatial win/stay task with the exception that animals could correct wrong choices. In the modified win/stay task, mice received an additional day of training (12 trials). Under these conditions, all mice improved performance during training [F_(11,209) _= 2.03; *p *< 0.05], but at no point during training did the KOs differ from the WTs in errors committed [F_(11,209) _= 1.07, n.s.]. Again, however, the KO mice took significantly longer navigating the maze to make correct arm choices [F_(11,209) _= 2.96; *p *< 0.001]. Again, these results suggest that KO animals require a longer time to execute ultimately accurate decisions under the demands of spatial navigation.

## Discussion

PEA-15 is a small phosphoprotein that binds to ERK and regulates ERK signaling [2]. In particular, PEA-15 can regulate activation of RSK2 and subsequent activation of CREB-mediated transcription [5]. The physiological and developmental function of PEA-15 is obscure. We now report that PEA-15 null mice exhibit cognitive deficiencies that are specifically limited to spatial learning tasks. Other forms of learning do not appear to be affected in any obvious way. The PEA-15 null mice also exhibit reduced nocturnal activity and complex changes in behavior related to exploration of stress- or anxiety provoking environments. Although KO mice exhibit weak grip strength, they appear to have normal visual, auditory, and olfactory abilities. Since several of the learning tasks in which KO mice exhibit normal performance can provoke stress or anxiety (e.g., fear conditioning and passive avoidance), the learning deficits that were consistently observed on spatial tasks are unlikely mediated by elevated stress reactivity or anxiety in these mice. Thus it appears that PEA-15 may play a critical role in the mediation of spatial learning, possibly via its enrichment in the hippocampus.

PEA-15 binds tightly to ERK and RSK2 and thereby regulates the outcome of ERK signaling [2,4]. More recently we have found that PEA-15 forms a complex with both ERK and RSK2 and is essential for ERK activation of RSK2 [5]. In fact, PEA-15 enhances RSK2 activation of CREB transcription. Thus in PEA-15 null mice, CREB activity is presumed to be reduced. This could account for the resultant spatial learning defects. We are currently testing this hypothesis in more detail.

Mutations in RSK2 cause the X-linked Coffin-Lowry syndrome [27]. Aside from skeletal and muscular defects, male patients also suffer from cognitive disabilities as determined by very low intelligence quotients (IQ<70) [22,28]. Mice that are RSK2 null also have learning disabilities though they appear less severe than those of Coffin-Lowry patients. Like PEA-15 null mice, the RSK2 nulls have impaired spatial learning and changes in exploratory behavior [23,29]. Because we see decreased activity of RSK2 in our PEA-15 null mice [5] we propose that the spatial learning disability they exhibit is the result of this reduced RSK2 activity. It may be that people with related cognitive problems, but who have normal RSK2, may have mutations in PEA-15. This remains to be determined. We should note that the published analysis of Coffin-Lowry learning deficiencies does not break down learning tasks to examine memory or tasks that might be related to spatial learning in particular. So it is unclear how the deficits in spatial learning in mice might relate to learning deficiencies of human patients.

Recently, morphometric analysis by MRI of Coffin-Lowry patients showed reduced cerebellum and hippocampus volumes [30]. However, no differences in brain size or morphology have yet been reported for RSK2 null mice. Moreover, we and others have not identified any differences in the brain size or morphology of the PEA-15 null mice [3]. Detailed analysis of the hippocampus of PEA-15 null mice has not revealed any differences in cellular morphology or size. Because of this we suspect that the learning disabilities in mice are due to differences in ERK MAP kinase signal transduction and CREB activation in the cells of the hippocampus rather than changes in its morphology.

In most respects the learning disabilities of PEA-15 null mice are similar to those of mice with impaired ERK activity. However, we found no significant change in fear conditioning in the KO mice, whereas there is literature reflecting a role for ERK in this process [31,32]. It may be that this is a result of where in the defect in the MAP kinase pathway is located. ERK can activate both ELK-1 and CREB (via RSK2), whereas RSK2 can activate only CREB. Thus it may be that the fear conditioning defects observed in the ERK experiments arise from decreased ELK-1 activity, while the changes in spatial learning may stem from changes in CREB activity. This remains to be tested. Alternatively, the differences in fear conditioning may arise from differences in the methods used.

We report that PEA-15 deficient mice exhibit an impaired ability at spatial learning tasks. They also have weaker forepaws than their WT counterparts do. It is therefore possible that the reduced forepaw strength may contribute to the performance of the mice in the spatial learning tests. However, in the spatial water maze, swim speed and path length analysis suggest that the reduced forearm strength in KO mice was not responsible for their impaired performance. The fact that KO mice did not display impaired basal activity in sensory motor tests also supports the premise that the learning impairments in spatial tasks were not contingent on gross differences in sensory/motor function between PEA-15 null and WT mice. In other learning and sensory/motor tests, the PEA-15 null mice showed no deficiencies. Thus the spatial learning defect is most likely independent of the differences in forepaw strength.

Our analysis was performed on adult mice in which PEA-15 had been completely deleted. The defects we observe could therefore be due to changes arising from lack of PEA-15 function in early brain development, lack of PEA-15 metabolic function in the behaving adult, or both. The current study cannot distinguish between these possibilities. However, this work provides the framework for additional studies that could address this issue. For example, we could create conditional knock-outs of PEA-15 in the brain in which PEA-15 is deactivated in the adult brain only after development is complete. These mice could then be run through a similar battery of tests to determine whether the effects are due to effects on brain development or adult brain function. These experiments are currently being planned.

## Conclusion

The ability to learn is a critical trait of many organisms [33] and the elucidation of the molecular mechanisms that mediate learning could have tremendous significance. Here we demonstrate that deletion of the PEA-15 gene caused impaired spatial learning in tasks that depend heavily on the hippocampus, and that this deficit was independent of typical performance confounds such as stress reactivity and emotionality. Thus PEA-15 may play a critical role in the mediation of spatial learning, possibly via its enrichment in the hippocampus. The molecular analysis of PEA-15 function in the brain may therefore supply new insights in our understanding of basic mechanisms of learning.

## Methods

### Subjects

PEA-15 null mice (KO; n = 13) and their wild-type controls (WT; n = 9) were obtained courtesy of H. Chneiweiss (College de France) and were generated by D. Kitsberg and P. Leder by homologous recombination as described [3]. In brief, clones containing the PEA-15 gene were selected from SV129 genomic library and were used to construct a targeting vector. The targeting vector, pNT, was constructed to delete a 4.5 kb BamHI-EcoRI genomic fragment that contains PEA-15 gene in its entirety. ES cells with the mutation were microinjected into C57BL/6J blastocytes and the embryos were implanted into pseudopregnant Swiss Webster female mice [3]. Subsequently, heterozygous females were serially backcrossed with C57BL/6J males to establish the PEA-15 null mice in the C57BL/6J background. Mice were genotyped by PCR and confirmed by immunoblotting for PEA-15 expression. KO and WT mice used in this study were 3-4-month-old males. In several instances, equipment malfunction or scheduling errors resulted in the failure to collect data from as many as two animals as noted. Thus the degrees of freedom associated with several statistical tests reflect fewer than the 22 subjects that served in this experiment.

Animals were singly housed in clear boxes with floors lined with wood shavings in a humidity- and temperature-controlled vivarium adjacent to testing rooms. A 12 hr/12 hr light/dark cycle was maintained. All behavioral training took place during the middle seven hours of the light cycle. Mice were handled in accordance with National Institutes of Health guidelines for the care and use of animals. All experiments were IACUC approved by the Institutional Review Board of Rutgers, The State University of New Jersey.

### General Behavioral Training and Testing Methods

All experiments were done blind to the genotype. For the learning tasks that required food deprivation, *ad lib *food was removed from the animals' home cages at the end of the light cycle approximately 40 hours prior to the start of training (and thus encompassing the "rest" day between successive tasks). During the deprivation period, animals were provided with food in their home cages for 60 min/day during the last 2 hrs of the light cycle, and thus were approximately 16 hrs food-deprived at the time of training or testing. Between each successive test (of learned and unlearned behaviors), animals received a day of rest. Different experimenters (n = 3) trained or tested animals in different tasks, and no experimenter was aware of animals' performance on other tasks, or genotype, until after the completion of the entire battery of tests. In total, animals were assessed in 11 tests of unlearned behavior and fitness, and seven tests of learning. In many of these tasks, multiple measures of performance were obtained such that a total of 12 measures of learned behavior and 25 measures of unlearned performance are reported.

### Tests of Unlearned Behaviors and Fitness

All animals were tested on 11 tasks that provided 25 measures of unlearned behaviors and/or fitness. A description of these tests and their implementation has been previously reported [34,35].

**1**. *Body Weight and Food Consumption under Mild Deprivation*. The body weights during periods of free feeding were collected every week throughout this series of tests, and the percent change in body weight was recorded after 24 hrs of food deprivation (prior to testing in the Lashley Maze).

**2**. *Running Wheel*. Animals were housed for 48 hours in cages containing running wheels in the home colony room. Animals were introduced to the running cages at the start of the light cycle on Day 1, and wheel cycles were recorded for 3 hrs as an index of running during initial adaptation. Total cycles on Days 2 and 3 and the percentage of cycles during the light and dark periods were counted.

**3**. *Elevated Plus Maze*. The maze was constructed of black Plexiglas in the form of a "plus". Each arm of the maze is 6 cm wide, and the maze is suspended 30 cm above a black surface. 8 cm high, black Plexiglas walls surrounded two opposing arms of the maze, and two of the arms were open. The maze was located in a 300 Lux environment. Animals were placed in the center of the maze facing an open arm, and their behavior in the maze was recorded in 1 min blocks for 4 min.

**4**. *Open Field Exploration*. A square field (46 × 46 cm) with 13 cm high walls of white Plexiglas was utilized. The apparatus was located in a brightly lit room (400 Lux) with a background noise of 65 dB. The field was conceptually divided into a grid comprised of 6 × 6 7.65 cm quadrants, where 20 of the quadrants abutted the outer walls of the field (i.e., "wall" quadrants), and 16 quadrants were displaced from the walls and comprised the interior (i.e. "open" quadrants) of the field. Animals were placed in the center of the field. After 20 sec had elapsed (during which the animals self-selected a starting location), the animals' behavior was monitored for 4-min. Throughout this time the animal's entries into wall and open quadrants were recorded. An entry was recorded whenever both front paws crossed the border of a quadrant.

**5**. *Straight Alley Exploration/Magnitude of Escape Response*. A straight alley was used that was 30 cm above ground. The alley was 244 cm long and 7 cm wide with 3 cm high walls. The initial 29 cm of the alley was enclosed in 12 cm high walls and an orange acetate ceiling. This portion was designated as the "start box" and the exit from this box could be blocked with a sliding guillotine door made of clear Plexiglas. The interior of the start box was 4 Lux, and the alley beyond the start box was 20 Lux. A startle stimulus could be delivered in the start box. This stimulus was the compound of a bright light (400 Lux) and a high-speed (3000 RPM) fan positioned so that its airflow was directed across the animal and down the alley. The fan raised background noise 50 dB. Animals were placed in the start box with the exit blocked. After 60 sec, the door was raised and animals were allowed to explore the alley for 4 min. The latency for each animal to cross a point in the alley 213 cm from the exit of the start box was recorded as were crossings across the midline of the alley. Both served as an index of exploratory behavior. After 4 min, the animals were returned to the start box where they were again confined for 60 sec. Subsequently the door was raised. At the moment that each animal moved to within 2 cm of the exit and faced the open alley, the compound startle stimulus was initiated and presented for 800 msec. This stimulus typically elicited rapid running (an "escape" response). The distance that the animal ran prior to making a complete stop of forward movement for at least 500 msec was recorded.

**6**. *Pain Sensitivity*. Upon being placed on a 52.6°C aluminum plate, animals' latency to raise a hind paw and to either lick or shake the paw served as the index of pain sensitivity.

**7**. *Balance Beam*. Animals were placed on a 40 × 0.7 × 2 cm (l × w × h) beam suspended 30 cm above the ground. In a 4 min test, mice exhibited wide variability in the amount of movement along its length.

**8**. *Roto-Rod Suspension*. Animals were hung by their front paws from a slowly rotating (8 RPM) rod (1 cm diameter, covered in hard rubber) suspended 30 cm above ground. Latency to drop from the rod (an index of grip strength) was recorded.

**9**. *Balance platform*. All four paws of animals were placed on a 3 cm round platform (60 Lux illumination) 30 cm above the ground. Latency to fall (240 sec maximum test duration) from the platform was recorded.

**10**. *Screen Climbing and Hanging*. Animals were placed on the underside of a wire mesh screen tilted 45° from vertical and suspended 24 cm from ground. The distance moved prior to dropping from the screen (cm/sec; 180 sec maximum test duration) and the latency to drop from the screen was computed.

**11**. *Black/White Preference*. A 10 × 36 cm chamber divided along its length in two equal halves was utilized. One half was white and brightly lit (100 Lux), and the other half was black and dim (5 Lux). A center wall with a 3 cm square opening that joins the black and white sides divided the two halves. Animals were placed in the black side of the chamber and allowed to explore for 4 min. The latency to first entry into the white chamber, percent of total time in the white chamber, and number of crossings between the black and white chambers was recorded.

### Tests of Learning

All animals were tested on seven learning tests, three of which could be solved most efficiently using a spatial strategy. The remaining four tasks are widely asserted to represent different, distinct learning domains, as summarized in Table 1. All tests are described in detail below.

**Table 1:** Task variables summary

**TABLE 1**	**Process**	**Test Stimulus**	**Motor** **Requirement**	**Organic Deprivation**	**Reinforcer**
	
**1. Lashley Maze**	operant approach	egocentric/visual	ambulation	food	BioServ Pellet (+)
	
**2. Passive Avoidance**	operant avoidance	place	passivity	none	noise/light (-)
	
**3. Spatial Water Maze**	operant escape spatial navigation	extramaze/visual	swimming	none	water immersion (-)
	
**4. Odor Discrimination**	discrimination	olfactory	ambulation	food	rice (+)
	
**5. Fear Conditioning**	association-formation	auditory	suppression	water	foot shock (-)
	
**6. Reinforced Alternation**	pattern recognition	prior choice/working memory	ambulation	food	cereal (+)
	
**7. Spatial Plus Maze**	operant approach/spatial navigation	extramaze/visual	ambulation	food	chocolate (+)

#### Odor Discrimination and Choice

In a procedure based on one designed by Sara [36] for rats, mice learned to navigate a square field in which unique odor-marked (e.g., almond, lemon, mint) food cups were located in three corners. Although food was present in each cup, it was accessible to the animals in only one cup (that marked by mint odor). An animal was placed in the empty corner of the field, after which it would explore the field and eventually retrieve the single piece of available food. On subsequent trials the location of the food cups was changed, but the accessible food was consistently marked by the same odor (i.e. mint).

A black Plexiglas 60 cm square field with 30 cm high walls was located in a dimly lit (40 Lux) testing room with a high ventilation rate (3 min volume exchange). Three 4 × 4 × 2.0 cm (l, w, h) aluminum food cups were placed in three corners of the field. A food reinforcer (30 mg portions of chocolate flavored puffed rice) was placed in a 1.6 cm deep, 1 cm diameter depression in the center of each cup. The food in two of the cups was covered (1.0 cm below the surface of the cup) with a wire mesh so that it was not accessible to the animal, while in the third cup (the "target" cup), the food could be retrieved and consumed.

A cotton-tipped laboratory swab, located between the center and rear corner of each cup, extended vertically 3 cm from the cups' surface. Immediately prior to each trial, fresh swabs were loaded with 25 ul of lemon, almond, or mint odorants (McCormick flavor extracts). The mint odor was always associated with the target food cup. In pilot studies, the odor associated with food was counterbalanced across animals, and no discernible differences in performance was detected in response to the different odors.

#### Lashley III Maze

The Lashley III maze consisted of a start box, four interconnected alleys, and a goal box containing a food reward. Over trials, the latency of rats to locate the goal box decreases, as do their errors (i.e., wrong turns or retracing). Here, the Lashley III maze was scaled for mice, and parameters were developed that supported rapid acquisition. The maze was constructed of black Plexiglas. A 2 cm wide × 0.1 cm deep white cup was located in the rear portion of the goal box, and 1/2 of a 45 mg BioServe (rodent grain) pellet served as reinforcers. Illumination was 80 Lux at the floor of the maze. The maze was isolated behind a shield of white Plexiglas to prevent extra-maze landmark cues.

Food-deprived animals were acclimated and trained on two successive days. On the day prior to acclimation, all animals were provided with three food pellets in their home cages to familiarize them with the novel reinforcer. On the acclimation day, each mouse was placed in the four alleys of the maze, but the openings between the alleys were blocked so that the animals could not navigate the maze. Each animal was confined to the start and subsequent two alleys for 4 min, and for 6 min in the last (goal) alley, where three food pellets were present in the food cup. On the training day, each animal was placed in the start box and allowed to traverse the maze until it reached the goal box and consumed the single food pellet present in the cup. Upon consuming the food, the animal was returned to its home cage for a 20 min interval (ITI), after which it was returned to the start box to begin the next trial. The apparatus was cleaned during each ITI, and the sequence was repeated for five trials. Both the latency and errors (i.e., a turn in an incorrect direction, including those which result in path retracing) to enter the goal box were recorded on each trial.

#### One-Trial Passive Avoidance

In order not to duplicate stimuli (i.e., shock) used to support associative learning (fear conditioning), we used a variant of the step-down avoidance task that does not rely on shock to motivate behavior. Upon stepping off the platform, animals were exposed to a compound of bright light and loud oscillating noise. A chamber illuminated by dim (< 5 Lux) red light was used for training and testing. Animals were confined to circular ("safe") chamber (10 cm diameter, 8 cm high). The walls and floor of this chamber were white, and the ceiling was translucent orange. The floor was comprised of plastic rods (2 mm diameter) arranged to form a pattern of 1 cm square grids. A clear exit door (3 CM square) was flush with the floor of the safe compartment, and the door could slide horizontally to open or close the compartment. The bottom of the exit door was located 4 cm above the floor of a second circular chamber (20 cm diameter, 12 cm high). This "unsafe" chamber had a clear ceiling and a floor comprised of 4 mm wide aluminum planks that formed a pattern of 1.5 cm square grids that were oriented at a 45° angle relative to the grids in the safe compartment. When an animal stepped from the safe compartment through the exit door onto the floor of the unsafe compartment, the compound aversive stimulus comprised of a bright (550 Lux) white light and oscillating ("siren") noise blast (2.4-3.7 kHz, 60 dBc above the 50 dBc background; Radio Shack Sound Oscillator, model 273-057) was initiated.

Animals were placed on the platform behind the exit blocked by the Plexiglas door. After 5 min of confinement, the door was retracted and the latency of the animal to leave the platform and make contact with the grid floor was recorded. Prior to training, step-down latencies typically range from 8-20 sec. Upon contact with the floor, the door to the platform was opened and the aversive stimulus (light+noise) was presented for 4 sec, at which time the platform door was opened to allow animals to return to the platform, where they were again confined for 5 min. At the end of this interval animals were returned to their home cages for a 1 hour retention period. Subsequently, mice were returned to the safe platform for 5 min, then the door was opened and the latency of the animal to exit the platform and step onto the grid floor (with no aversive stimulation) was recorded, completing training and testing. The ratio of post-training to pre-training step-down latencies were calculated for each animal and served to index learning.

#### Associative Fear Conditioning

Animals were exposed to a stimulus (i.e. a CS; tone) that terminated in the onset of a mild foot shock (i.e. a US). These tone-shock (CS-US) pairings came to elicit conditioned fear responses when animals were subsequently presented with the tone. To avoid any interaction of the training context (which itself acquires an association with shock) with the CS at the time of testing, training and testing were conducted in separate distinct contexts. Two distinct experimental chambers (i.e. contexts; 32 × 28 × 28 cm, l × w × h) were used, each of which was contained in a sound- and light-attenuating enclosure. These boxes were designated as "training" and "testing" contexts, and differ as follows: The training context was brightly illuminated (100 Lux), had clear Plexiglas walls, no lick tube, and parallel stainless-steel rods (5 mm, 10 mm spacing) forming the floor. The test context was dimly illuminated (6 Lux), the walls covered with an opaque pattern of alternating black and white vertical stripes (3 cm wide), and the floor was formed from stainless 1.5 mm rods arranged at right-angles to form a grid of 8 mm squares. A water-filled lick tube protruded through a small hole in one wall of the test chamber, such that the tube's tip was flush with the interior surface of the wall at a point 3 cm above the floor. Upon contacting the tube, the animal completed a circuit such that the number of licks/sec could be recorded. This circuit was designed so that if an animal made continuous contact with the tube (i.e. "mouthed" the tip), the circuit recorded eight licks/sec, a rate that approximates continuous licking. In the training chamber, a 0.6 mA constant-current scrambled footshock (US) could be delivered through the grid floor. In both the training and test chambers, a 40 dB above background white noise (the CS) could be presented through speakers mounted at the center of the chambers ceiling.

Water-deprived animals were acclimated to the training and test chambers by placing them each in both contexts for 20 min on the day prior to training. Training occurred in the training context in a single 30 min session during which each animal was administered a noise-shock pairing 10 and 20 min after entering the chamber. Each 10 sec noise terminated with the onset of a 500 msec footshock. At the end of the training session, animals were returned to their home cages for 60 min, after which they were re-acclimated to the test context for 20 min where they were allowed free access to the lick tubes. On the subsequent day (23-25 hours post training), animals were tested. Each animal was placed in the test context whereupon after making 25 licks, the noise CS was presented continuously until the animal completed an additional 25 licks. The latency to complete the last 25 licks during the pre-tone interval and in the presence of the tone was recorded, with an 800 sec limit imposed on the second 25 licks (a limit not reached by any animal described here). With these measures, the latency to complete 25 licks in the presence of the tone CS served as our index of learned fear, and the latency to complete 25 licks prior to CS onset served as an index of basal lick rates.

#### Spatial Water Maze

For this task, animals were introduced into a round pool of opaque water from which they could escape onto a hidden (i.e., submerged) platform. The latency for animals to find the platform decreases across successive trials. We have developed a protocol in which mice exhibit significant reductions in their latency to locate the escape platform within six training trials [34,37]. First, animals were confined in a clear Plexiglas cylinder on the safe platform for 5 min on the day prior to training. Second, a considerably longer ITI (10 min) was used than is typical (c.f., 90 sec). Lastly, the maze, surround, and water were black; visual cues were constructed of patterns of lights

A round black pool (140 cm diameter, 56 cm deep) was filled to within 24 cm of the top with water made opaque by the addition of nontoxic, water soluble, black paint. A hidden 11 cm diameter perforated black platform was in a fixed location 1.5 cm below the surface of the water midway between the center and perimeter of the pool. The pool was enclosed in a ceiling-high black curtain on which five different shapes (landmark cues) were variously positioned at heights (relative to water surface) ranging from 24-150 cm. Four of these shapes were constructed of strings of white LEDs (spaced at 2.5 cm intervals) and included an "X" (66 cm arms crossing at angles 40° from the pool surface), a vertical "spiral" (80 cm long, 7 cm diameter, 11 cm revolutions), a vertical line (31 cm) and a horizontal line (31 cm). The fifth cue was constructed of two adjacent 7 W light bulbs (each 4 cm diameter). A video camera was mounted 180 cm above the center of the water surface. These cues provided the only illumination of the maze, totaling 16 Lux at the water surface.

On the day prior to training, each animal was confined to the escape platform for 300 sec. Training was conducted on the two subsequent days. On Day 1 of training, animals were started from a unique location on each of five trials. An animal was judged to have escaped from the water (i.e., located the platform) at the moment at which four paws were situated on the platform, provided that the animal remained on the platform for at least 5 sec. Each animal was left on the platform for a total of 30 sec, after which the trial was terminated. Trials were spaced at 10 min intervals, during which time the animals were held in a warmed (27.5°C) opaque (5 Lux) box lined with wood shavings. On each trial, a 90 sec limit on swimming was imposed, at which time any animal that had not located the escape platform was placed by the experimenter onto the platform, where it remained for 30 sec. Animals were observed from a remote (outside of the pool's enclosure) video monitor, and the animals' performance was recorded on videotape for subsequent analysis. Day 2 of training proceeded as Day 1. However, after the last (fifth) training trial, a 2-hour retention period was begun, after which animals were tested with a "probe" trial. On the probe test, the escape platform was removed from the pool, and all animals were started from the sixth position for that day. A 60 sec test was conducted in which the animals' time searching in the target quadrant (that in which the escape platform was previously located) and non-target quadrants were recorded.

#### Spatial Plus Maze (WinStay)

An elevated maze in the form of a "+" was constructed of black Plexiglas, each of the four arms measuring with 8 × 40 cm (W × L). A 4 mm diameter food cup was located in the center of the arm 2 cm from its end. Food (a 20 mg chocolate-flavored Noyes Rodent Pellet) was located in every cup, but was accessible to the animal on in the arm designated as "west". Twenty-four cm from the end of each arm and equidistant between successive arms were 18 × 18 cm visual cues, a black (240 pt) "X", "O", and "+".

Animals were adapted to the maze on Day 1. They were placed in the north start box where they were held for 30 sec, released, and allowed to explore the unbaited maze for 3 min. This process was then repeated for the east and south arms. On Day 2, animals were trained, and a food reinforcer was present in the west arm on each trial. On the first two trials (Trial "0A" and "0B"), the animal was placed in the north start box for 30 sec, and was then released and allowed to explore the maze until consuming the food in the West arm. On subsequent trials, the animal was started in the east and then south arm, followed by the reverse order of arms until a total of 12 trials (Trials 1-12) were completed. On Trials 1-12, an entry into an incorrect arm terminated the trial (at which time the exit was blocked and the animal removed after 5 sec). If mice chose the baited arm they were allowed to consume the chocolate-flavored Noyes Rodent Pellet. An ITI of 60 sec in the home cage separated each trial. Animals' choices were recorded on each trial

#### Reinforced Alternation

An elevated maze in the form of a "T" was constructed of black Plexiglas. Each of the two cross- arms measured 36 cm in length, 4 cm in width, with 10 cm walls. A 4 mm diameter food cup was located in each cross-arm, 2 cm from its end. The base of the 'T' consisted of a 14 cm start box and a 16 cm central compartment from which the cross-arms connected. The portion designated as the "start box" could be blocked with a sliding guillotine door as can the intersection between each cross-arm and the central compartment. The maze was diffusely lit from above (80 Lux).

Food deprived mice learned to alternate arm choices in a "T-maze" to obtain food reinforcement. The number of training trials required before an animal learns to consistently alternate is an index of rate at which they learn the correct pattern. On the first day of training, animals were adapted to the maze, in which 1/16^th ^of a fruit loop (General Mills) was available at the end of each choice arm. Over four more trials, mice were forced to alternate arm entry by closing the opposite arms' guillotine door. In the forced choice arm animals obtained a reinforcer. On the subsequent day, animals were placed in the start compartment (at the base of the T), held behind the closed guillotine door for 60 sec, and then, after the door was opened, allowed to choose one arm for entry, wherein the reward was available. On subsequent trials (30 sec ITI), the animal could choose either arm, but food was available only in the arm opposite the arm reinforced on the prior trial. Incorrect choices terminated the trial. On ensuing trials, food was available in the same arm until a correct choice was made and the food was retrieved. With our adaptation and training procedures, young adult mice often begin to perform without error after 6-10 training trials. Training proceeded for 12 trials; the number of trials required by an animal prior to its initiating three consecutive correct choices was used as an index of that animal's performance.

### Statistical Analyses

Comparisons of groups were conducted with either one- or two-factor analyses of variance (ANOVA). In experiments where a single measure of a dependent variable (e.g., "Bolli Excreted", Fig 1B) was obtained, a one-way ANOVA was performed to determine if GROUPS differed. In experiments where a dependent variable was repeatedly assessed (e.g., "Weights", Fig 1A), a two-factor ANOVA was performed, where GROUP constituted one factor, and TIME OF TEST constituted the second factor. In this instance, the second factor represented a repeated measure, and thus the second factor was analyzed as a within-subjects variable. In the case of the two-factor ANOVA, each factor and the interaction of the factors were considered for statistical significance. In all cases, *p*s < 0.05 were considered significant.

## Authors' contributions

DAT, DP, SK, KL, and GH all assisted in the design of the experiments and conducted behavioral tests. JWR and LDM conceived the study and participated in the design and coordination of the study as well as drafted the manuscript. All authors read and approved the final manuscript.
